# Antarctic Genomics

**DOI:** 10.1002/cfg.398

**Published:** 2004-04

**Authors:** Melody S. Clark, Andrew Clarke, Charles S. Cockell, Peter Convey, H. William Detrich III, Keiron P. P. Fraser, Ian A. Johnston, Barbara A. Methe, Alison E. Murray, Lloyd S. Peck, Karin Römisch, Alex D. Rogers

**Affiliations:** 1 British Antarctic Survey National Environment Research Council High Cross Madingley Road Cambridge CB3 0ET UK; 2 Department of Biology Northeastern University Boston MA 02115 USA; 3 Gatty Marine Laboratory University of St Andrews St Andrews Fife KY Scotland 16 8LB UK; 4 TIGR 9712 Medical Center Drive Rockville MD 20850 USA; 5 Desert Research Institute 2215 Raggio Parkway Reno NV 89512 USA; 6 University of Cambridge CIMR and Department of Clinical Biochemistry Wellcome Trust/MRC Building Hills Road Cambridge CB2 2XY UK

## Abstract

With the development of genomic science and its battery of technologies, polar biology stands on the threshold of a revolution, one that will enable the investigation of important questions of unprecedented scope and with extraordinary depth and precision. The exotic organisms of polar ecosystems are ideal candidates for genomic analysis. Through such analyses, it will be possible to learn not only the novel features that enable polar organisms to survive, and indeed thrive, in their extreme environments, but also fundamental biological principles that are common to most, if not all, organisms. This article aims to review recent developments in Antarctic genomics and to demonstrate the global context of such studies.


**Comparative and Functional Genomics**
*Comp Funct Genom* 2004; **5**: 230–238.
Published online in Wiley InterScience (www.interscience.wiley.com). **DOI:** 10.1002/cfg.398

**Feature**

### Antarctic genomics

Melody S. Clark[1]*, Andrew Clarke[1], Charles S. Cockell[1], Peter Convey[1], H. William Detrich III[2], Keiron P. P. Fraser[1], Ian A. Johnston[3], Barbara A. Methe[4], Alison E. Murray[5], Lloyd S. Peck[1], Karin Römisch[6] and Alex D. Rogers[1]

[1] *British Antarctic Survey, National Environment Research Council, High Cross, Madingley Road, Cambridge CB3 0ET, UK*
[2] *Department of Biology, Northeastern University, Boston, MA 02115, USA*
[3] *Gatty Marine Laboratory, University of St Andrews, St Andrews, Fife KY16 8LB, Scotland, UK*
[4] *TIGR, 9712 Medical Center Drive, Rockville, MD 20850, USA*
[5] *Desert Research Institute, 2215 Raggio Parkway, Reno, NV 89512, USA*
[6] *University of Cambridge, CIMR and Department of Clinical Biochemistry, Wellcome Trust/MRC Building, Hills Road, Cambridge CB2 2XY, UK*

**Correspondence to: Melody S. Clark, British Antarctic Survey, High Cross, Madingley Road, Cambridge CB3 0ET, UK. E-mail: mscl@bas.ac.uk*

### Abstract

**With the development of genomic science and its battery of technologies, polar biology stands on the threshold of a revolution, one that will enable the investigation of important questions of unprecedented scope and with extraordinary depth and precision. The exotic organisms of polar ecosystems are ideal candidates for genomic analysis. Through such analyses, it will be possible to learn not only the novel features that enable polar organisms to survive, and indeed thrive, in their extreme environments, but also fundamental biological principles that are common to most, if not all, organisms. This article aims to review recent developments in Antarctic genomics and to demonstrate the global context of such studies. Copyright © 2004 John Wiley & Sons, Ltd.**

Received: 23 January 2004
Revised: 5 February 2004
Accepted: 11 February 2004

**Keywords: Antarctic; genomics; psychrophilic; stenothermal; marine; terrestrial; environment; evolution**

> Unplanned natural experiments create ecological communities that we would never have dreamed of creating (Diamond, 2001).

### Background

Genomics is high-profile science, impacting on all areas of biology, so it is not surprising that it is also playing an increasing role in Antarctic studies. The Antarctic is often viewed as one of Earth's last great frontiers; the extreme climate combined with months of complete or near darkness provide inhospitable conditions for life. Despite this, the evolutionary history and geographical isolation of the Antarctic has produced a unique environment, rich in species adapted to the extreme conditions. Genome mining is clearly high on the agenda and, although not without controversy (cf. Lake Vostok), it also provides unparalleled tools for studying natural selection in action and investigating the link between organisms and environment (environmental genomics). With the publication in 2003 of a US National Research Council position paper entitled 'Frontiers in Polar Biology in the Genomic Era' (NRC 2003) and a recent workshop meeting on Polar Genomics at the British Antarctic Survey, it seems timely to review progress so far and discuss the prospects for the future.

### The evolution of the Antarctic environment

The evolution of the Antarctic flora and fauna has been influenced by geological and climatic factors including geographic isolation of the continent and shelf seas, extreme low temperatures and strong seasonality. The Antarctic continent

Copyright © 2004 John Wiley & Sons, Ltd.

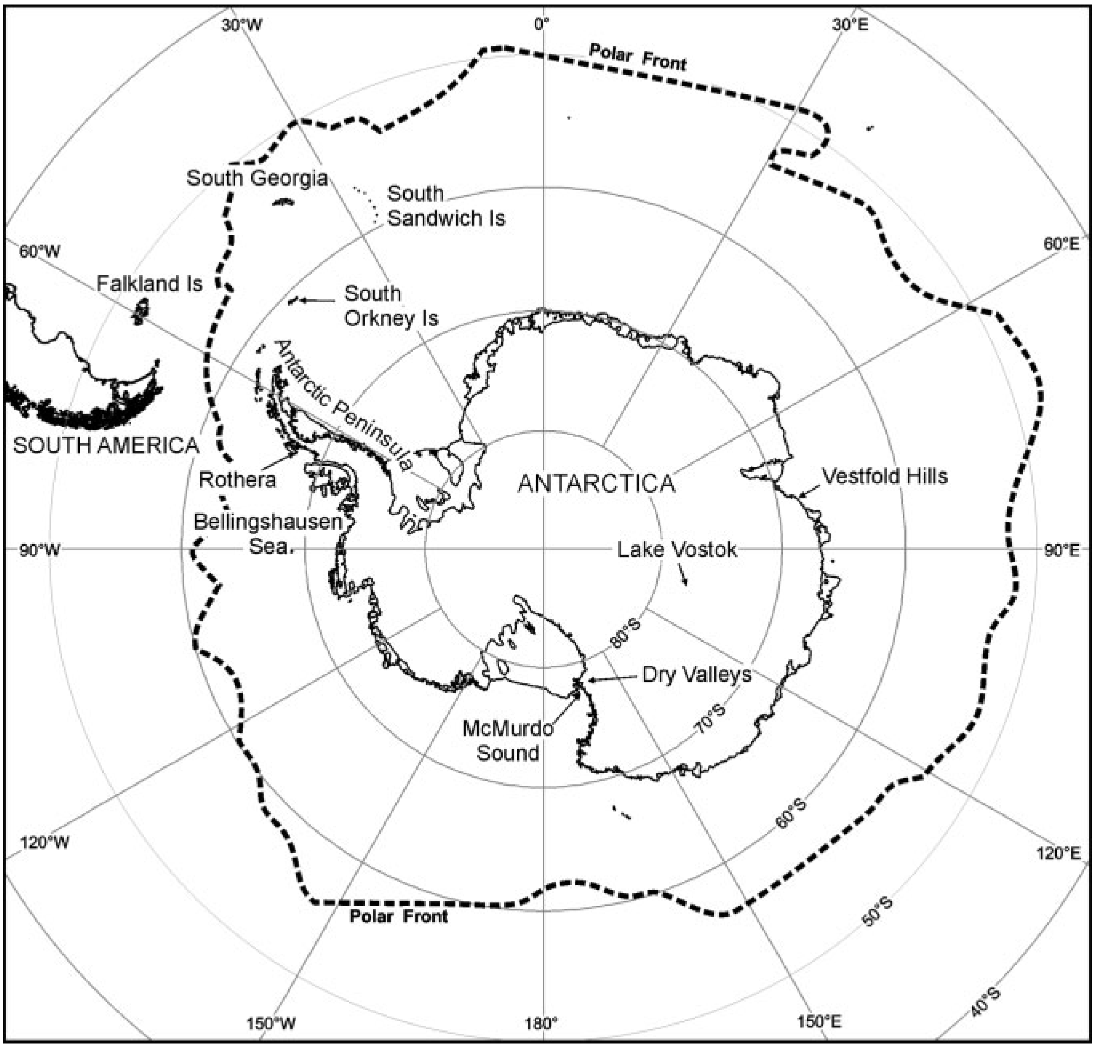

**Figure 1.** Map of Antarctica and the Southern Ocean

(Figure 1) emerged from the disintegration of the Gondwana supercontinent as a result of tectonic forces in the Cretaceous period (120 million years ago). The continent became more isolated through time, finally separating from South America about 33.7 million years ago. This time also marked the onset of major cooling in the Antarctic with the first appearance of sea ice (Clarke and Crame, 1992). Since then Antarctic temperatures have generally decreased, but the trend has been punctuated by episodes of warming that were global, or localized to the Antarctic.

Many groups of organisms became extinct in the Antarctic as a result of the increasingly extreme climate, although this is likely to have taken place episodically over the last 30 million years rather than as a single event. Examples include decapod crustaceans and most groups of fish in the marine environment, whilst higher plants are absent from the land, with the exception of two species that are found in the maritime Antarctic (Antarctic Peninsula and islands in the Southern Ocean). The terrestrial fauna has an extremely low diversity with some soil communities being the simplest on Earth. Much of the coastal flora and fauna may have arisen from the relatively recent immigration of species as most, if not all, of this habitat was destroyed by recent glaciations (Convey, 2001). There is evidence, however, that the continental Antarctic biota may have survived

Copyright © 2004 John Wiley & Sons, Ltd. 

glaciations in refugia and some elements represent ancient Gondwanan relicts (e.g. Marshall and Pugh, 1996). Additionally there is evidence that some groups of animals have invaded the Antarctic during warm periods and subsequently speciated *in situ* (e.g. Page and Linse, 2002).

The contrast in environmental variability in terrestrial versus marine systems has led to markedly different evolutionary pressures on organisms in these two ecosystems. In the most southerly marine areas, organisms may only experience temperature variations of <0.5 °C annually. In contrast, terrestrial organisms can be exposed to daily temperature fluctuations of 20–40 °C and annual temperature fluctuations as high as 40–80 °C (Convey, 1996). Terrestrial species also face long periods of restricted resource availability in winter because the environment is frozen. These conditions have prevailed in Antarctica for at least 10 million years, a long evolutionary period and longer than the glaciation of high northern latitudes. Given this unique evolutionary history and current environmental setting, the Antarctic biota provides many opportunities to address fundamental biological problems, in particular the links from genome to survival of organisms and the functioning of ecosystems.

### Antarctic fish as model organisms

Among polar organisms, the phylogenetic history of the Antarctic fishes of the suborder Notothenioidei (Teleostei) is undoubtedly the best understood (Near *et al.*, 2003). Living in a stable, extremely cold and well-oxygenated marine environment, these fishes have evolved numerous adaptive changes in their biochemical and physiological functions. These compensatory adaptations have involved both the alteration of existing genes to produce enzymes that function well at cold temperatures and the restructuring of the genome to gain new physiological capabilities. In recognition of this potential, NHGRI funding has been granted for the creation of two BAC libraries for *Chaenocephalus aceratus* and *Notothenia coriiceps* (**http://www.genome.gov/10001852**). For details of these libraries contact either H. William Detrich III (**iceman@neu.edu**) or Chris Amemiya (**camemiya@benaroyaresearch.org**).

One major example of these adaptations is the acquisition of genes for antifreeze glycoproteins by recruitment and modification of existing genomic 'raw material', in the form of a pancreatic trypsinogen-like gene (Cheng and Chen, 1999). By contrast, living in a stable, cold environment has also led to the loss of capabilities normally thought to be essential to vertebrate life. Examples of this include the heat-shock response in at least one Antarctic fish. (Hofmann *et al.*, 2000) and myoglobin in the cardiac muscle in six of the fifteen members of the icefishes (Channichthyidae) (Moylan and Sidell, 2000). The latter are the most derived family of the notothenioid suborder and they possess one of the most unusual of these phenotypes; the forfeiture of the respiratory oxygen transporter haemoglobin and the ability to produce functional erythrocytes (di Prisco *et al.*, 2002). Scanning the genomes and transcriptomes of red-blooded notothenioids for genes that are no longer expressed by the natural icefish 'erythroid knockout' model will help to elucidate the genetic programme of erythropoiesis in all vertebrates, including humans. Undoubtedly, the functions of novel erythroid genes discovered using the Antarctic fish system will be characterized most efficiently by cloning and analysing their orthologues in more tractable vertebrate genetic models, e.g. the zebrafish and mouse (so-called 'model-hopping'). These discoveries may ultimately be exploited to produce new treatments for blood-related diseases and syndromes, such as anaemias associated with kidney dialysis treatment and cancer chemotherapy.

The relatively high oxygen concentration possible in water at low temperatures has also allowed the evolution of giant muscle fibres in some notothenioids (Johnston *et al.*, 2003). The increase in muscle fibre size is possible because diffusional constraints are relaxed, and is associated with a dramatic reduction in muscle fibre number for a given body size. There are defined differences documented between the development of muscle fibres in some Antarctic fish and most other fish species (Johnston *et al.*, 2003). This presents ideal opportunities for the application of subtractive genomics techniques to discover novel genes associated with the genetic mechanisms controlling fibre number. The study of such processes is of considerable economic importance for farmed species such as salmon, trout and cod, because a high muscle fibre

Copyright © 2004 John Wiley & Sons, Ltd.

number is associated with improved flesh quality, particularly firmness and processing characteristics (Johnston, 2001).

Some species derived from the core radiation of Antarctic notothenioids have subsequently invaded more temperate waters, including representatives of the genus *Harpagifer* in the Falkland Islands and South America and *Paranotothenia* in New Zealand. Comparative genomic studies of these closely related (congener) species provide opportunities for investigating critical genetic adaptations associated with cold adaptation and also the loss/gain of function mutations described above.

### Analysis of cold adaptation

### Antarctic fish

Clearly, Antarctic fish present several candidate models for exploitation in the medical and agricultural fields of science, but they are also of use in more fundamental research, dissecting enzymic adaptation and polypeptide folding. Both tubulin dimers and the microtubule motors of Antarctic fish are cold-adapted; their properties at 0 °C are similar to organisms living at much warmer temperatures. Cold adaptation has been shown to be the result of small changes in primary sequences and post-translational modifications, including hydrophobic remodelling and increased flexibility of the domains involved in dimer–dimer contact (Detrich *et al.*, 1998, 1992). Similar changes producing increases in molecule flexibility have also been identified in the $A_4$-lactate dehydrogenases from a range of different fish species, including notothenioids (Fields and Somero, 1998). Such studies are enhanced by examining enzyme orthologues in a range of congener species showing different degrees of temperature adaptation (Fields *et al.*, 2002). Dissecting the mechanisms leading to conformation changes in proteins provides an insight, not only into the general principles behind the evolutionary adaptation of organisms, but also into polypeptides that adopt multiple conformations, such as pathogenic prion proteins in mammals (Cohen and Prusiner, 1998).

There is also an industrial interest in dissecting the nature of protein function at low temperatures. For example, the improvement of the secretory capacity of cells used in industry has been a long-standing goal that has remained largely elusive as a result of our limited understanding of the principles governing protein folding and secretion. Recently, the genes encoding the core component of the protein translocation channel in the endoplasmic reticulum (ER) membrane, *SEC61*, have been sequenced from Antarctic and Arctic fishes and compared to their mesophilic counterparts (Römisch, 2003). The results indicate that the Antarctic toothfish (*Dissostichus mawsoni*) ER imports proteins with high efficiency at 0 °C, lending support to the hypothesis that the protein translocation machinery in these organisms is adapted to low temperature (Römisch, 2003). It is now possible to introduce the corresponding amino acid changes into the protein translocation channel of a genetically tractable model organism, such as *Saccharomyces cerevisiae*, or into tissue culture cells (disabling the endogenous channel by RNA interference), and directly observe their effects on protein translocation across the ER membrane at various temperatures.

### Microorganisms

One of the constraints on the study of Antarctic fish species is the current lack of sequence data, although this is beginning to be addressed with the development of BAC libraries. However, this is not such a problem with Antarctic microorganisms and their small genomes, which are more amenable to exploitation. This is one area in which genomics is creating an immediate impact through the analysis of genome sequences from cold-adapted bacteria and Archaea (Table 1) and the ability to compare them to their mesophilic and thermophilic counterparts.

Insights into thermal adaptation came through comparative studies of the draft genomes of two methanogenic Archaea, *Methanogenium frigidum* and *Methanococcoides burtonii*, from Ace Lake in the Vestfold hills region of Antarctica (Saunders *et al.*, 2003), with 19 other archaeal genomes representing organisms with optimal growth rates in the range 15–98 °C. Psychrophilic (thriving at temperatures below 20 °C) methanogen proteins contained structural features thought to confer molecular flexibility, which increase catalytic efficiency and prevent cold denaturation. In addition, both genomes contained cold shock domains or folds that have putative roles in RNA stabilization. Among the

Copyright © 2004 John Wiley & Sons, Ltd. 

**Table 1.** Status of sequenced psychrophilic microorganisms

| Organism | Origin of strain | Status of genome sequence | Location of project |
|---|---|---|---|
| *Methanogenium frigidum* | Ace Lake, Antarctica | Draft sequence published, Saunders *et al.* 2003 | Amersham Biosciences, USA; Australian Genome Research Facility, Australia |
| *Methanococcoides burtonii* DSM6242 | Ace Lake, Antarctica | Draft sequence published, Saunders *et al.* 2003 | JGI, USA |
| *Cenarchaeum symbiosum** | Symbiont marine sponge (*Axinella mexicana*) off California coast | In annotation | JGI, USA |
| *Colwellia psychrerythraea* 34H | Arctic marine sediments | Manuscript in preparation | TIGR, USA |
| *Vibrio salmonicida* | Farmed Atlantic salmon, coast of Norway | In progress | University of Tromso, Norway |
| *Photobacterium profundum* SS9 | Amphipod homogenate from 2.5 km deep in the Sulu Sea | In annotation | CRIBI Biotechnology Centre, Italy |
| *Shewanella violacea* DSS12 | Deep-sea mud (5.1 km) of Ryukyu trench, Japan | Manuscript in preparation | IAB, Keio University, Japan |
| *Psychrobacter* sp. 273–4 | Siberian permafrost Arctic marine sediments | In annotation | JGI, USA |
| *Desulfotalea psychrophila* LSv54 | Arctic marine sediments, Svalbard | Manuscript in preparation | MPI, Germany |
| *Exiguobacterium* 255-15 | Siberian permafrost | In annotation | JGI, USA |
| *Flavobacterium psychrophilum* | Salmonoid pathogen, Montana fish hatchery | In progress | NCCCWA, USA |
| *Polaribacter filamentus* | Surface seawater, 350 km north of Deadhorse, Alaska | Draft sequence completed | Integrated Genomics, USA |

* The true psychrophilic nature of *C. symbiosum* remains unknown, since the strain is uncultivated, although it lives in waters at 10–15 °C. Data from these projects can be BLAST-searched at: JGI (Joint Genome Institute) via **http://www.jgi.doe.gov/JGI_microbial/html/index.html;** TIGR (The Institute for Genomic Research) via **http://www.tigr.org/tdb/mdb/mdbinprogress.html;** the *Desulfotalea* genome, MPI (Max Planck Institute) can be accessed via **http://www.regx.de/blast/index.html;** and the *M. frigidum* genome can be accessed at **http://psychro.bioinformatics.unsw.edu.au/blast/mf_blast.php**

most important adaptations to the cold are changes in microbial cell membranes, such as the production of polyunsaturated fatty acids to maintain fluidity at low temperatures, and synthesis of enzymes capable of catalysis at low temperatures (Deming, 2003). Cold-adapted enzymes are already being utilized in industries and products as diverse as leather tanning, food processing and in laundry detergent for cold water washing of clothes (Feller and Gerday, 2003).

The completion of the *Colwellia psychrerythraea* genome provides one of the first whole genomes of a strictly psychrophilic bacterium (Methe *et al.*, 2002). As a member of the $\gamma$-proteobacteria, the genus *Colwellia* represents a group of obligate marine psychrophiles whose members to date have been obtained exclusively from cold environments from Arctic, Antarctic deep oceans, sea water, sea ice and sediment, in which they play important roles in carbon and nutrient cycling. Recent biochemical investigations have demonstrated that *C. psychrerythraea* is capable of producing extracellular proteases with extraordinarily low temperature activity optima (Huston *et al.*, 2000). In addition, as a member of the $\gamma$-proteobacteria it is closely related to several mesophiles whose genomes have also been completed. Thus, analysis of the *C. psychrerythraea* genome provides an excellent opportunity to utilize comparative genomics approaches to related mesophiles, including members of the genera *Shewanella* and *Vibrio*.

Whilst genome analysis can facilitate the discovery of novel proteins and secondary metabolites, the elucidation of probable metabolic pathways or metabolic potential of each microorganism is fundamental to understanding its role in biogeochemical cycling. This information in turn

Copyright © 2004 John Wiley & Sons, Ltd. 

is critical to the additional applications of microorganisms as potential agents of wastewater treatment and bioremediation of toxic pollutants in cold environments (including temperate environments that experience a winter season).

### Ecosystem monitoring

Linkages between gene, genome content, gene regulation and expression can provide information about Antarctic terrestrial and marine biodiversity and ecosystem function. Antarctica harbours an incredible variety of environments dominated by microorganisms, ranging from sea ice, the water column and benthos in the marine ecosystem, to volcanoes, rocks and soils, and all the different forms of ice and water on the continent (Table 2). Of those habitats investigated, molecular biological approaches have been useful in a number of instances to describe the diversity of the inhabitants. These tools are a great benefit since, as in many other environments on earth, our abilities are limited for cultivating the majority of, or the ecologically relevant, microorganisms (Amann *et al.*, 1995), with slow growth rates at low temperatures experienced in the Antarctic being an added challenge.

Sequences that are phylogenetically informative in bacteria, such as ribosomal RNA (rRNA), have been used to describe significant temporal shifts in bacterioplankton community composition in both the marine and freshwater ecosystem over the annual cycle (Church *et al.*, 2003; Murray *et al.*, 1999; Pearce, 2003). This ecological pattern is probably linked to the ecosystem role that the Archaea play, although this role remains unknown as yet. The presence of gene products or of genes themselves can reflect organism responses to ecosystem changes. Booth *et al.* (2001) showed that plankton in surface waters receive a damaging dose of radiation because of higher than normal UV-B exposure in late spring, resulting from the ozone hole. DNA repair, evidenced by detection of *rec*A mRNA and the RecA protein, increased throughout the day and was at low levels during the night, suggesting that DNA repair follows the daily 24 h cycle and is an important aspect of microbial survival in Antarctic surface waters.

**Table 2.** Antarctic habitats give refuge to a host of microorganisms, many of which still remain unknown, yet contain a wealth of genomic information potentially revealing keys to survival in extremes of cold, dessication, salt, UV and low nutrients. In addition, their metabolites and enzymes hold potential for novel uses in both health and industrial applications of biotechnology

| Marine | Terrestrial (aquatic) | Terrestrial |
|---|---|---|
| Sea ice | Ponds | Snow |
| Water column | Perennially ice-covered lakes | Ice sheets |
| Sediments | Permanently ice-covered lakes | Glaciers |
| Sea ice brine | Subglacial lakes | Soils |
| Marine invertebrates | Lake ice | Volcanos |
| Marine vertebrates | Rivers and streams | Rocks |

Environmental genomics approaches based on random cloning of large fragments of DNA from the environment have recently been used to reveal genome content and microheterogeneity in marine plankton (Beja *et al.*, 2002). A similar approach confirmed the presence of several large genomic clones containing the gene for proteorhodopsin in Antarctic waters, a pigment involved in a recently described novel type of photoheterotrophy in the ocean (De La Torre *et al.*, 2003). For these groups of organisms, and many of those that are thought to be the most abundant in marine ecosystems that have not been cultivated to date, the environmental genomics approach holds promise in helping make key connections between the organisms, their metabolic capabilities and adaptations to the Antarctic ecosystem.

### Antarctic species as models for dissecting environmental stress responses

The environmental extremes experienced in Antarctica are not unique in the world, e.g. low temperatures comparable with those of the maritime Antarctic can be experienced in New York. The scientific value of Antarctica lies in the *combination* of a set of environmental stressors and the insights that those stresses may give us about the genomic responses to environmental extremes elsewhere on the planet. On land, extreme low temperatures, desiccation, freezing, osmotic stress and elevated UV radiation are some of the main environmental stressors to be found on the continent. In the sea, extreme seasonality, freezing and physical disturbance from ice are the main stressors.

Physiological studies of organisms give the impression that the responses to these different

Copyright © 2004 John Wiley & Sons, Ltd.

extremes may be mediated through different pathways. The field response of the Antarctic terrestrial alga *Prasiola crispa* to UV and freezing were studied over a 13-month period (Jackson and Seppelt, 1997). Concentrations of proline, an amino acid that assists in osmotic adjustment, were found to be increased in response to freezing, and concentrations of methanol-extracted UV-screening compounds increased during months of elevated UV exposure. Davey (1989) also suggested that cellular response to freezing was different to that for dehydration in the same species (*P. crispa*). Investigations carried out on the effects of environmental stresses on the extremophilic cyanobacterium *Chroococcidiopsis* sp., found in the McMurdo Dry Valleys, however, showed that in this organism an increase in temperature could synergistically cause an increase in the production of the UV-screening compound, scytonemin, alongside increases in UV radiation (Dillon *et al.*, 2002). This suggests that the pathways for extreme temperature and UV radiation responses might in some way be linked.

Although physiological studies provide tantalizing insights into the possible overlap of pathways used in response to different extremes, only by examining the expression of the genes themselves under different stresses is it possible to determine which suites of genes have a common role in different stresses and unravel how these different stresses are linked. There are global implications in such studies; some of the combinations of stresses experienced in the Antarctic are similar to extreme environments in other regions of the world. For example, desiccation and extreme UV radiation can be experienced by organisms in hot deserts near the equator, albeit at high temperatures. Furthermore, studies of genomic responses to combined stresses will yield important insights into resource allocation. Depending on the different extremes experienced, organisms must allocate their energy reserves to the different responses required (Convey, 2001). Overlap of genetic responses may allow for a more efficient response to multiple stressors. This should be important to organisms that have limited reserves or nutrient availability, which is often the case in Antarctica and other extreme deserts. Finally, by investigating the expression of specific genes, it will be possible to derive fundamental insights into how these responses are similar, or vary, in quite different phylogenies, from microorganisms to fish, and to what extent extreme environments require specific adaptations or simply select for more generalist or phenotypically plastic life-styles.

### Antarctic species as primary monitors of global climate change effects

Global temperatures increased by $0.6 \pm 0.2\,°C$ during the 20th century, probably as a result of anthropogenic increases in greenhouse gases (Houghton *et al.*, 2001). On a regional scale, however, the picture is more complex, e.g. in the Antarctic, the air temperature of the Peninsula has increased by 2 °C over the last 40–50 years (King and Harangozo, 1998). The mean winter water temperatures of lakes on Signy Island (Maritime Antarctic) have also increased, by 0.9 °C between 1980 and 1995 — three to four times faster than global mean temperature increases; as a result, permanent lake ice cover has reduced by 45% (Quayle *et al.*, 2002).

Terrestrial communities are already exposed to large seasonal and annual variations in temperature and are therefore highly eurythermal (Convey *et al.*, 2003). They are more likely to be able to tolerate regional increases in air temperature, as predicted global temperature increases are small compared to the thermal ranges many of these species experience (Convey, 2003). They are already experiencing changes in populations and habitat ranges (Convey, 2003) and the localized retreat of some glaciers and permanent ice (Smith, 1990) will offer opportunities for colonization of pristine habitats in newly exposed areas. This will present a novel chance to study genome function and evolution in pioneer species.

In contrast, Antarctic marine organisms are highly stenothermal, in response to stable water temperatures. At McMurdo Sound mean annual water temperatures are $-1.8\,°C$ with a standard deviation of only 0.2 °C, while at the more northerly Rothera Research Station on the Antarctic Peninsula (Figure 1), water temperatures only vary by 2–3 °C throughout the year (Clarke and Leakey, 1996). Some marine species have experimental upper temperatures below 5 °C and long-term upper lethal temperature limits of only $+2\,°C$, and are therefore extremely vulnerable to any rapid increase in seawater temperatures (Peck, 2002a). Evidence suggests that Antarctic marine ectotherms

Copyright © 2004 John Wiley & Sons, Ltd. 

are living close to the limits of their metabolic scopes, and this limits their ability to cope with elevated temperatures (Peck, 2002b; Pörtner 2002). Unlike the other continents, there are no long coastlines spanning a wide range of environmental conditions in Antarctica that allow migration away from deteriorating conditions.

Genomic techniques provide the ability to detect indications of thermal stress in organisms significantly earlier than would be possible with physiological measurements alone. Understanding the molecular mechanisms employed by a range of Antarctic organisms in coping with increased environmental temperature will enable predictions to be made as to how they and other Antarctic species will adapt to global climate change, in terms of physiological function, distribution patterns and ecosystem balance. Additionally they will provide an early understanding of these processes in other regions of the world, which are more complex systems, and which are subject to other human influences, such as pollution. With the present disparity in climate change in different regions of the Antarctic, an ideal opportunity exists to evaluate genomic responses in organisms affected and unaffected by climate warming. Temperate congeners of Antarctic species can then be used to place the responses found in Antarctic species in a global context.

### Concluding remarks

The application of genomics and functional genomics approaches to the examination of Antarctic organisms is still in its infancy. In spite of this, benefits are already being produced in the form of insights into complex biological processes and biotechnological exploitation. Future years will see an increase in the production of genomic resources and sequence data, which will greatly facilitate such studies, providing information across the whole range of biological levels from DNA to ecosystem. The Antarctic represents a natural freezer to which the door has remained closed for 30 million years — perhaps the door is about to open.

### Acknowledgements

Supported in part by US National Science Foundation Grant No. OPP-0089451 (to H.W.D.). I.A.J. supported by NERC Grant No. GR3/12550. K.R. supported by Wellcome Trust Grant No. 042216. This paper was produced by BAS staff within the LATEST and ABPPF core programmes.

### References

Amann R, Ludwig W, Schleifer KH. 1995. Phylogenetic identification and *in situ* detection of individual microbial cells without cultivation. *Microbiol Rev* **59**: 143–169.

Beja O, Koonin EV, Aravind L, *et al.* 2002. Comparative genomic analysis of archaeal genotypic variants in a single population and in two different oceanic provinces. *Appl Environ Microbiol* **68**: 335–345.

Booth MG, Hutchinson L, Brumsted M, *et al.* 2001. Quantification of recA gene expression as an indicator of repair potential in marine bacterioplankton communities of Antarctica. *Aquat Microb Ecol* **24**: 51–59.

Chen C-HC, Chen L. 1999. Evolution of an antifreeze glycoprotein: a blood protein that keeps Antarctic fish from freezing arose from a digestive enzyme. *Nature* **401**: 443–444.

Church MJ, DeLong EF, Ducklow HW, *et al.* 2003. Abundance and distribution of planktonic Archaea and Bacteria in the waters west of the Antarctic Peninsula. *Limnol Oceanogr* **48**: 1893–1902.

Clarke A, Crame JA. 1992. The Southern Ocean benthic fauna and climate change: a historical perspective. *Phil Trans R Soc B Biol Sci* **338**: 299–309.

Clarke A, Leakey RJG. 1996. The seasonal cycle of phytoplankton, macronutrients and the microbial community in a nearshore Antarctic marine ecosystem. *Limnol Oceanogr* **41**: 1281–1294.

Cohen FE, Prusiner SB. 1998. Pathological conformations of prion proteins. *Annu Rev Biochem* **67**: 793–819.

Convey P. 1996. The influence of environmental characteristics on life history attributes of Antarctic terrestrial biota. *Biol Rev* **71**: 191–225.

Convey P. 2001. Terrestrial ecosystem responses to climate changes in the Antarctic. In *'Fingerprints' of Climate Change*, Walther GR, *et al.* (eds). Kluwer: New York; 17–42.

Convey P. 2003. Maritime Antarctic climate change: signals from terrestrial biology. In *Antarctic Peninsula Climate Variability: a Historical and Palaeoenvironmental Perspective*, Domack E, Burnett A, Leventer A, *et al.* (eds). Antarctic Research Series No. 79. American Geophysical Union: Washington; 45–158.

de la Torre JR, Christianson LM, Beja O, *et al.* 2003. Proteorhodopsin genes are distributed among divergent marine bacterial taxa. *Proc Natl Acad Sci USA* **100**: 12 830–12 835.

di Prisco G, Cocca E, Parker S, Detrich HW III. 2002. Tracking the evolutionary loss of hemoglobin expression by the white blooded Antarctic icefishes. *Gene* **295**: 185–191.

Davey MC. 1989. The effects of freezing and desiccation on photosynthesis and survival of terrestrial Antarctic algae and cyanobacteria. *Polar Biol* **10**: 29–36.

Deming JW. 2003. Psychrophiles and polar regions. *Biofutur* **229**: 43–50.

Detrich HW III. 1998. Molecular adaptation of microtubules and microtubule motors from Antarctic fish. In *Fishes of Antarctica*, de Prisco G, Pisano E, Clarke A (eds). Springer: London: 139–149.

Detrich HW III, Fitzgerald TJ, Dinsmore JH, Marchese-Ragona SP. 1992. Brain and egg tubulins from Antarctic fishes

Copyright © 2004 John Wiley & Sons, Ltd. 

are functionally and structurally distinct. *J Biol Chem* **267**: 18 766–18 775.

Diamond J. 2000. Ecology. Damned experiments! *Science* **294**: 1847–1848.

Dillon JG, Tatsumi CM, Tandingan PG, Castenholz RW. 2002. Effect of environmental factors on the synthesis of scytonemin, a UV-screening pigment, in a cyanobacterium. *Arch Microbiol* **177**: 322–331.

Feller G, Gerday C. 2003. Psychrophilic enzymes — hot topics in cold adaptation. *Nature Rev Microbiol* **1**: 200–208.

Fields PA, Kim Y-S, Carpenter JF, Somero GN. 2000. Temperature adaptation in *Gillichthys* (Teleostei: Gobiidae) $A_4$-lactate dehydrogenases: identical primary structures produce subtly different conformations. *J Expt Biol* **205**: 1293–1303.

Fields PA, Somero GN. 1998. Hot spots in cold adaptation: localized increases in conformational flexibility in lactate dehydrogenase A4 orthogs of Antarctic notothenioid fishes. *Proc Natl Acad Sci USA* **95**: 11 476–11 481.

Fowbert JA, Lewis Smith RI. 1994. Rapid population increases in native vascular plants in the Argentine Islands, Antarctic Peninisula. *Arc Alp Res* **26**: 290–296.

Hofmann GE, Buckley BA, Airaksinen S, *et al.* 2000. Heat-shock protein expression is absent in the Antarctic fish *Trematomus bernacchii* (Family Nototheniidae). *J Exp Biol* **203**: 2331–2339.

Houghton JT, Ding Y, Griggs DJ, *et al.* 2001. *Climate Change 2001: the Scientific Basis. Contribution of Working Group I to the Third Assessment Report of the Intergovernmental Panel on Climate Change*. Cambridge University Press: Cambridge.

Huston AL, Krieger-Brockett BB, Deeming JW. 2000. Remarkably low temperature optima for extracellular enzyme activity from Arctic bacteria and sea ice. *Environ Microbiol* **2**: 383–388.

Jackson AE, Seppelt RD. 1997. Physiological adaptations to freezing and UV radiation exposure in *Prasiola crispa*, an Antarctic terrestrial alga. In *Antarctic Communities: Species, Structure and Survival*, Battaglia B, Valencia J, Walton DWH (eds). University of Cambridge: Cambridge.

Johnston IA. 2001. Implications of muscle growth patterns for the colour and texture of fish flesh. In *Farmed Fish Quality*, Kestin SC, Warriss PD (eds). Blackwell Scientific: Oxford; 13–30.

Johnston IA, Fernández DA, Calvo J, *et al.* 2003. Reduction in muscle fibre number during the adaptive radiation of notothenioid fishes: a phylogenetic perspective. *J Exp Biol* **206**: 2595–2609.

King JC, Harangozo SA. 1998. Climate change in the western Antarctic Peninsula since 1945: observations and possible causes. *Ann Glaciol* **27**: 571–575.

Marshall DJ, Pugh PJA. 1996. Origin of the inland acari of continental Antarctica, with particular reference to Dronning Maud Land. *Zool J Linn Soc* **118**: 101–118.

Methe B, Lewis M, Weaver B, *et al.* 2002. The *Colwellia* strain 34H genome sequencing project. Abstract 142 of the DOE Ninth Genome Sequencing Contractor–Grantee Workshop, 27–31 January, Washington, DC.

Moylan TJ, Sidell BD. 2000. Concentrations of myoglobin and myoglobin mRNA in heart ventricles from Antarctic fishes. *J Exp Biol* **203**: 1277–1286.

Murray AE, Wu KY, Moyer CL, Karl DM, DeLong EF. 1999. Evidence for circumpolar distribution of planktonic Archaea in the Southern Ocean. *Aquat Microb Ecol* **18**: 263–273.

Near TJ, Pesavento JJ, Cheng CH. 2003. Mitochondrial DNA, morphology, and the phylogenetic relationships of Antarctic icefishes (Notothenioidei: Channichthyidae). *Mol Phylogenet Evol* **28**: 87–98.

NRC. 2003. *Frontiers in Polar Biology in the Genomic Era*. National Academy Press: Washington, DC (**http://www.nap.edu/catalog/10623.html**).

Page T, Linse K. 2002. More evidence of speciation and dispersal across the Antarctic Polar Front through molecular systematics of Southern Ocean *Limatula* (Bivalvia: Limidae). *Polar Biol* **25**: 818–826.

Pearce DA. 2003. Bacterioplankton community structure in a maritime Antarctic oligotrophic lake during a period of holomixis, as determined by denaturing gradient gel electrophoresis (DGGE) and fluorescence *in situ* hybridisation (FISH). *Microbial Ecol* **46**: 92–105.

Peck LS. 2002a. Ecophysiology of Antarctic marine ectotherms: limits to life. *Polar Biol* **25**: 31–40.

Peck LS. 2002b. Coping with change: stenothermy, physiological flexibility and environmental change in Antarctic seas. Proceedings of the 14th International Congress on Comparative Physiology. **www.liv.ac.uk/ciliate/climate/peck.html**

Pörtner HO. 2002. Climate variations and the physiological basis of temperature dependent biogeography: systematic to molecular hierarchy of thermal tolerance in animals. *Comp Biochem Physiol A* **132**: 739–761.

Pörtner HO, Peck LS, Zielinski S, Conway LZ. 1999. Intracellular pH and energy metabolism in the highly stenothermal Antarctic bivalve *Limopsis marionensis* as a function of ambient temperature. *Polar Biol* **22**: 17–30.

Quayle WC, Peck LS, Peat H, *et al.* 2002. Extreme responses to climate change in Antarctic lakes. *Nature* **295**: 645.

Römisch K, Collie N, Soto N, *et al.* 2003. Protein translocation across the endoplasmic reticulum membrane in cold-adapted organisms. *J Cell Sci* **116**: 2875–2883.

Saunders NFW, Thomas T, Curmi PMG, *et al.* 2003. Mechanisms of thermal adaptation revealed from the genomes of the Antarctic Archaea *Methanogenium frigidum* and *Methanococcoides burtonii*. *Genome Res* **13**: 1580–1588.

Smith RIL. 1990. Signy Island as a paradigm of biological and environmental change in Antarctic terrestrial ecosystems. In *Antarctic Ecosystems — Ecological Change and Conservation*, KR Kerry, G Hempel (eds). Springer-Verlag: Berlin; 32–50.

Copyright © 2004 John Wiley & Sons, Ltd. 
